# Circulating miRNAs are associated with successful bone regeneration

**DOI:** 10.3389/fbioe.2025.1527493

**Published:** 2025-03-28

**Authors:** Julia K. Frank, Carina Kampleitner, Patrick Heimel, Gabriele Leinfellner, Dominik Hanetseder, Simon Sperger, Amelie Frischer, Barbara Schädl, Stefan Tangl, Claudia Lindner, Johanna Gamauf, Regina Grillari-Voglauer, Fergal J O’Brien, Marianne Pultar, Heinz Redl, Matthias Hackl, Johannes Grillari, Darja Marolt Presen

**Affiliations:** ^1^ Herz Jesu Krankenhaus, Vienna, Austria; ^2^ Ludwig Boltzmann Institute for Traumatology, The Research Center in Cooperation with AUVA, Vienna, Austria; ^3^ Austrian Cluster for Tissue Regeneration, Vienna, Austria; ^4^ Karl Donath Laboratory for Hard Tissue and Biomaterial Research, University Clinic of Dentistry, Medical University of Vienna, Vienna, Austria; ^5^ University Clinic of Dentistry, Medical University of Vienna, Vienna, Austria; ^6^ Evercyte GmbH, Vienna, Austria; ^7^ Tissue Engineering Research Group, Department of Anatomy and Regenerative Medicine, Royal College of Surgeons in Ireland, Dublin, Ireland; ^8^ Trinity Centre for Biomedical Engineering, Trinity Biomedical Sciences Institute, Trinity College Dublin, Dublin, Ireland; ^9^ Advanced Materials and Bioengineering Research (AMBER) Centre, Royal College of Surgeons in Ireland & Trinity College Dublin, Dublin, Ireland; ^10^ TAmiRNA GmbH, Vienna, Austria; ^11^ Institute of Molecular Biotechnology, Department of Biotechnology, BOKU University, Vienna, Austria; ^12^ Centre for the Technologies of Gene and Cell Therapy, The National Institute of Chemistry, Ljubljana, Slovenia

**Keywords:** gene-activated scaffold, osteogenesis, bone regeneration, extracellular vesicles, biomarkers, circulating microRNAs

## Abstract

**Introduction:**

Bone healing is a well-orchestrated process involving various bone cells and signaling pathways, where disruptions can result in delayed or incomplete healing. MicroRNAs (miRNAs) are small non-coding RNAs capable of influencing various cellular processes, including bone remodeling. Due to their biological relevance and stable presence in biofluids, miRNAs may serve as candidates for diagnosis and prognosis of delayed bone healing. The aim of the study was to investigate changes in miRNAs circulating in the blood during the healing of rat calvaria defects as biomarkers of successful bone regeneration.

**Methods:**

Standardized calvaria defects were created in 36 Wistar rats with a trephine drill and treated with collagen hydroxyapatite (CHA) scaffolds. The treatment groups included CHA scaffolds only, CHA scaffolds containing a plasmid coding for bone morphogenetic protein 2 (BMP2) and miR-590-5p, CHA scaffolds containing mesenchymal stromal cell-derived extracellular vesicles, and empty defects as a control group. After 1, 4 and 8 weeks of healing, the animals were evaluated by microcomputed tomography (microCT), as well as subjected to histological analyses. Blood was sampled from the tail vein prior to surgeries and after 1, 4, and 8 weeks of healing. miRNAs circulating in the plasma were determined using next-generation sequencing.

**Results:**

Variability of bone regeneration within the four groups was unexpectedly high and did not result in significant differences between the groups, as indicated by the microCT and histological analyses of the newly formed bone tissue. However, irrespective of the treatment group and regenerative activity, we identified miRNAs with distinct expression patterns of up- and downregulation at different time points. Furthermore, rats with high and low regenerative activity were characterized by distinct circulating miRNA profiles. miR-133-3p was identified as the top upregulated miRNA and miR-375-3p was identified as the top downregulated miRNA in animals exhibiting strong regeneration over all time points evaluated.

**Conclusion:**

Our study indicates that regardless of the treatment group, success or lack of bone regeneration is associated with a distinct expression pattern of circulating microRNAs. Further research is needed to determine whether their levels in the blood can be used as predictive factors of successful bone regeneration.

## 1 Introduction

Bone has an intrinsic capacity for regeneration as part of the repair process in response to a trauma, as well as during skeletal development and remodeling in adult life (Einhorn, 1998; Dimitriou et al., 2011). However, disruptions in regenerative processes can result in delayed or incomplete healing. Studies identifying biomarkers to monitor bone defect regeneration are still scarce (Hollinger and Leong, 1996; Agrawal and Ray, 2001; Ho-Shui-Ling et al., 2018; Nambiar et al., 2022). Therefore, specifically minimally-invasive surrogate markers of bone healing are of high interest to support the stratification of fracture healing into early, delayed, or failed healing, with high-risk of non-unions (Ueda et al., 2003; Tabata, 2009; Cunniffe et al., 2010; Komatsu et al., 2016; Levingstone et al., 2016) MicroRNAs (miRNAs) have evolved as important regulators of multiple biological pathways. Due to their small size, active or passive release, and association with protein complexes, miRNAs are relatively resistant to extracellular degradation and can be isolated from different liquid biopsies, including blood. miRNAs circulating in the blood are considered very promising for diagnosis and prognosis of diseases and have already been proposed as biomarkers in various contexts of bone pathologies including osteoporosis, delayed bone healing (Hackl et al., 2016; Wang et al., 2018), and rare bone diseases (Gao et al., 2022).

But to our knowledge, few or no studies have demonstrated their potential to evaluate or even predict the success of bone regenerative strategies.

In the current study, we aimed to compare side by side two different strategies of augmenting collagen-hydroxyapatite (CHA) scaffolds to enhance bone regeneration (Raftery et al., 2015; Raftery et al., 2018), and at the same time evaluate profiles of miRNA circulating in the blood at different timepoints of healing. In the first approach, plasmid DNA (pDNA) was used in combination with CHA scaffolds to enhance bone regeneration (Lyons et al., 2010; Tierney et al., 2012; Murphy et al., 2014). It was previously shown that CHA scaffolds loaded with bone morphogenetic protein 2 (BMP2) plasmid, optimized for therapeutic protein expression, enhanced the regeneration of rat calvaria defects (Hacobian et al., 2016; Raftery et al., 2018; Brenner et al., 2021). Similarly, Mencía Castaño et al. (2016) have previously demonstrated that CHA scaffolds incorporating nano-hydroxyapatite complexes as non-viral delivery vehicles for therapeutic antagomiR-133a effectively augmented osteogenesis mediated by human mesenchymal stem cells. Here, we evaluated the pDNA containing expression cassettes for BMP2 and microRNA 590-5p, found in our previous study to enhance osteogenic differentiation (Brenner et al., 2021). In the second approach, we loaded CHA scaffolds with extracellular vesicles (EVs) derived from mesenchymal stromal cells (MSCs) (Gantenbein et al., 2020; O’Brien et al., 2020), as prior studies showed that MSC-derived EVs can enhance bone regeneration in a rat model of nonunion bone regeneration (Qin et al., 2016; Zhang et al., 2016; Zhang et al., 2020).

## 2 Material and Methods

### 2.1 Study protocol

The study protocol was approved by the local Committee for Animal Experiments of the City Government of Vienna (MA58-821/18). A total of 36, 12 weeks old male Sprague- Dawley rats (Janvier Labs, Le Genest-Saint-Isle, France) with a body weight of 430–500 g at the time of surgery were used for four treatment groups as described in the following sections. The animals were treated according to the guidelines for animal care with free access to water and a standard diet.

### 2.2 Scaffold fabrication and loading with extracellular vesicles and therapeutic plasmid DNA

CHA scaffolds were manufactured by a freeze-drying process as reported previously (O’Brien et al., 2007) and cross linked dehydrothermally (DHT) at 105 °C for 24 h at 0.05 bar in a vacuum oven (Vacucell 22; MMM, Germany), followed by chemical cross-linking using a mixture of 6 mM N-(3-Dimethylaminopropyl)-N′-ethyl carbodiimide hydrochloride (EDC) and 5.5 mM NHydroxysuccinimide (NHS). Scaffolds were cut into disks with a diameter of 8 mm and a height of 1.5 mm to fit the defect size and reflect the thickness of native calvaria bone.

EVs produced from hTERT-immortalized Wharton’s jelly-derived mesenchymal stromal cells (WJ-MSC/TERT273) using a hollow fiber bioreactor (Fibercell Systems Inc., New Market, United States) and enriched using tangential flow filtration were loaded on each CHA scaffold as described in the following section.

Gene activated scaffolds were produced using the hybrid BMP2 and miR-590-5p plasmid described previously by Brenner et al. (2021). BMP-2 release and efficacy were extensively characterized by Hacobian et al. (2016) *in vitro* (2D) and by Raftery et al. (2015), Raftery et al. (2018) *in vitro* and *in vivo* (3D).

pDNA was complexed with 3Dfect (OZbiosciences, San Diego, United States) according to the manufacturer’s protocol. To reduce animal stress and ensure comparability with pDNA treatments, a one-time treatment approach was used. Study of Brancolini et al. (Brancolini et al., 2024) supports the functionality of WJ-EVs in anti-inflammatory, anti-fibrotic, and wound healing contexts.

### 2.3 Calvaria defect model

Rats received pre-surgical analgesia by oral (p.o.) administration of meloxicam (1 mg/kg, Boehringer Ingelheim Vetmedica GmbH, Ingelheim, Germany). Thereafter, rats were subjected to 4%–6% sevoflurane (Sevoflurane, Baxter, Vienna, Austria) to induce general anesthesia, followed by an intraperitoneal injection of 60 mg/kg ketamine hydrochloride (Ketamidor, Richter Pharma, Wels, Austria) and 0.5 mg/kg medetomidine (Domitor, Orion Corporation, Espoo, Finland). Following anesthesia, the skin of the head was shaved and cleaned using 70% ethanol and povidone iodine solution before the animal was placed on a heated aseptic platform. After a midsagittal incision over the calvarium, the periosteum was completely removed. In each animal, one central circular defect was created using a trephine drill with a diameter of 8 mm (Stoma, Emmingen-Liptingen, Germany) under constant irrigation and with special care to preserve the underlying dura mater. The animals were randomly assigned to four treatment groups: (a) empty defect (E); (b) non-augmented CHA scaffolds (S); (c) CHA scaffolds containing 2 µg plasmid encoding for bone morphogenetic protein 2 (BMP2) and miR-590-5p (gene activated scaffold) (GS); (d) CHA scaffolds loaded with 25 µL in of EVs at a concentration of 2 × 10^11^ particles per ml PBS (EV). Scaffolds were placed into the defects, whereas untreated, empty defects served as a control. Wounds were closed with resorbable sutures (Vicryl 5–0; Ethicon GmbH, Norderstedt, Germany) and 0.5 mg/kg atipamezole (Antisedan, Orion Corporation) was injected subcutaneously (s.c.) to reverse the sedative effect of medetomidine. For pain relief, buprenorphine (0.05 mg/kg, s. c., dosage interval: 8 h; Richter Pharma AG) and meloxicam (1 mg/kg, p. o., once daily; Boehringer Ingelheim Vetmedica GmbH) were administered for 72 h. To avoid an infection at the surgical site, enrofloxacin (10 mg/kg, s. c.; Bayer, Leverkusen, Germany) was injected once daily for 96 h. The animals were sacrificed after 8 weeks of healing by an intracardial overdose of thiopental (Sandoz GmbH, Vienna, Austria), and tissues were harvested and fixed in 4% neutral-buffered formalin.

### 2.4 Blood collection

Blood was sampled from the tail vein prior to surgery (week 0), 1, 4, and 8 weeks after surgery. The animals were subjected to inhalation anesthesia using 3%–6% sevoflurane in oxygen and the blood was sampled into K2-EDTA Eppendorf tubes from the tail vein. Samples were then centrifuged at 2500 g for 15 min at 20°C and the plasma was separated and stored at −80°C. On completion of the study, the 6 best and 6 worst healing rats were chosen according to the microcomputed tomography (microCT) measurement values of bone volume (marked in Supplementary Figure S1), and the plasma samples for each of the four timepoints (week 0, 1, 4, 8) were collected.

### 2.5 Microcomputed tomography analysis

*In vivo* microCT scans were performed at 1, 4 and 8 weeks after surgery (at the same time as the blood sampling) under general anesthesia as described in the previous section using a SCANCO vivaCT 75 device (SCANCO Medical AG, Brüttisellen, Switzerland) with 70 kVp, 116 μA, performing 720 projections/180° with an exposure time of 225 ms. Scans were reconstructed to an isotropic resolution of 50 µm. Week 1 was thresholded differently to capture calcification of early bone for bone volume.

To confirm the *in vivo* microCT findings, *ex vivo* microCT scans were performed only at 8 weeks after euthanasia in a SCANCO µCT 50 device (SCANCO Medical AG, Brüttisellen, Switzerland) with 90 kVp, 200 μA, performing 850 projections/180° with an integration time of 250 ms and hardware binning 2. Scans were reconstructed to an isotropic resolution of 20.7 µm.

MicroCT measurements were performed on the *in vivo* CT scans using Fiji software (Schindelin et al., 2012). Scans were rotated, so that the defect was aligned to the XY plane. A circle selection with a diameter of 8 mm was centered on the defect defining the region of interest (ROI). Bone volume (BV) was measured in the ROI with a threshold of 200 mgHA/cm^3^ (at week 1) and 400 mgHA/cm^3^ (at week 4 and 8). A maximum intensity projection was created of the segmented defect and the relative area of bone in the projection was measured as the percent coverage of the defect. The thickness of the bone structures was calculated using the Local Thickness Fiji software (Schindelin et al., 2012).

### 2.6 Histological analysis

Twelve fixed tissue samples were selected from the four treatment groups reflecting strong (n = 6) and weak (n = 6) calvarial bone regeneration according to microCT measurements and dehydrated in ascending grades of ethanol and embedded in light-curing resin (Technovit 7200 VLC +1% benzoyl peroxide, Kulzer and Co., Wehrheim, Germany). Blocks were further processed using the EXAKT cutting and grinding equipment (Exakt Apparatebau, Norderstedt, Germany). Uncalcified thin-ground sections were prepared perpendicular to the sagittal suture through the center of the defect and were stained with Levai-Laczko dye for descriptive histology. In this staining, newly formed bone appears dark pink, mature lamellar bone light pink, osteoid unstained and soft tissue blue. The sections were scanned using an Olympus BX61VS digital virtual microscopy system (DotSlide 2.4, Olympus, Japan, Tokyo) with a ×20 objective resulting in a resolution of 0.32 μm per pixel.

### 2.7 Circulating microRNA analysis

#### 2.7.1 Total RNA extraction

For total RNA extraction we standardized the input volume to 80 µL plasma, which were initially added to 120 µL nuclease-free water (NFW) to achieve a total input volume of 200 µL for RNA extraction. This was performed using the miRNeasy mini kit (Qiagen, Germany), by homogenizing the sample with 1 mL of Qiazol to which 1 µL of miRCURY spike-in mix (Qiagen, Germany) was added to monitor RNA extraction efficiency. Chloroform extraction, precipitation, washing and elution in 30 µL NFW was performed as described previously (Kocijan et al., 2020).

#### 2.7.2 Small RNA-sequencing

Small RNA-sequencing was performed using the miND^®^ workflow as described previously (Khamina et al., 2022). Briefly, 8.5 µL of total RNA were mixed with 1 µL miND^®^ spike-ins and used as input for library preparation using a single-adapter ligation protocol (Barberán-Soler et al., 2018) based on the RealSeq Biofluids kit (RealSeq Biosciences, US). Following adapter ligation, circularization, and reverse transcription, libraries were amplified by PCR (21 cycles) introducing a unique index primer sequence with each library. Library concentration and size were controlled using capillary electrophoresis (Agilent Bioanalyzer DNA 1000) and equimolar amounts based on the miRNA library concentration at 144 bp were pooled. The final pool was size purified to remove adapters and primers using the BluePippin 3% Agarose Cassettes (SageScience, US). The resulting library was sequenced on an Illumina NovaSeq 6000 SP1 (100 cycles, single-end) flow cell. Raw data were demultiplexed on the instrument and subject to further data analysis using the miND^®^ pipeline (Diendorfer et al., 2022).

#### 2.7.3 Small RNA-sequencing data analysis

Overall quality of the next-generation sequencing data was evaluated automatically and manually with fastQC v0.11.9 (Andrews, 2010) and multiQC v1.10 (Ewels et al., 2016). Further data analysis was performed using the miND^®^ pipeline (Diendorfer et al., 2022): reads from all passing samples were adapter trimmed and quality filtered using cutadapt v3.3 (Martin, 2011) and filtered for a minimum length of 17 nt. Mapping steps were performed with bowtie v1.3.0 (Langmead et al., 2009) and miRDeep2 v2.0.1.2 (Friedländer et al., 2012), whereas reads were mapped first against the genomic reference Rnor.6.0 provided by Ensembl (Zerbino et al., 2018) allowing for two mismatches and subsequently miRBase v22.1 (Griffiths-Jones, 2004), filtered for miRNAs of rno only, allowing for one mismatch. For a general RNA composition overview, non-miRNA mapped reads were mapped against RNAcentral (Sweeney et al., 2019) and then assigned to various RNA species of interest. Statistical analysis of preprocessed NGS data was done with R v4.0 and the packages pheatmap v1.0.12, pcaMethods v1.82 and genefilter v1.72. Differential expression analysis with edgeR v3.32 (Robinson et al., 2010) used the quasi-likelihood negative binomial generalized log-linear model functions provided by the package. The independent filtering method of DESeq2 (Love et al., 2014) was adapted for use with edgeR to remove low abundant miRNAs and thus optimize the false discovery rate (FDR) correction. Results were considered as significant at FDR <0.05. Small RNA-sequencing raw data have been deposited at the NCBI Gene Expression Omnibus (GEO) archive under the accession ID GSE279239.

### 2.8 Statistical analysis of imaging parameters

For this study, a total of 36 rats were divided into four treatment groups which were originally of equal sample size (n = 9). However, 6 animals had to be excluded from the study due to severe problems during or shortly after the surgical procedure (n = 5) or an incomplete *in vivo* microCT scan (n = 1) resulting in the following allocation: empty defect (n = 7), CHA scaffold (S; n = 8), CHA gene-activated scaffold (GS; n = 7), CHA extracellular vesicle scaffold (EV; n = 8).

Data representation and statistical analysis of the measured microCT parameters were performed using Prism 10.0 (GraphPad Software, Boston, United States). Data are shown as a combination of box plots with median and range and individual values. Normal data distribution was evaluated, and statistical differences between treatment groups were determined using a nonparametric Kruskal–Wallis test with Dunn’s *post hoc* test for multiple comparison. Differences were considered statistically significant when p < 0.05. Histological analyses are presented in a descriptive manner.

### 2.9 Target prediction analysis

Target prediction for individually selected miRNAs was performed using the tool miRNAtap v1.40.0 (Maciej Pajak, 2017) allowing to combine the results from 5 most commonly cited prediction algorithms: DIANA (Maragkakis et al., 2009), Miranda (Enright et al., 2003), PicTar (Lall et al., 2006), TargetScan (Friedman et al., 2009), and miRDB (Wong and Wang, 2015). Predictions were filtered to include only those identified in at least three databases. Gene ontology (GO) enrichment analysis was performed for individual gene targets as well as for the combined prediction results. Hyper-geometric testing, using all human genes as reference dataset, was used to derive enriched biological processes. GO enrichment analysis was conducted using the R packages org. Hs.e.g.,.db v3.20.0 and topGO v2.58.0 (Adrian Alexa, 2017). Results are visualized using ggplot2 v3.5.1 and chorddiag v0.1.3.

## 3 Results

### 3.1 Microcomputed tomography and histological analyses identify animals exhibiting strong and weak regeneration responses regardless of the treatment modality

Calvaria defect healing was evaluated in a total of 30 rats after 1, 4 and 8 weeks of healing using *in vivo* microCT imaging (Supplementary Figure S1). The treatment groups included non-augmented CHA scaffolds (group S), CHA scaffolds loaded with plasmid encoding for BMP2 and miR-590-5p (group GS) and scaffolds loaded with Wharton’s jelly MSC-EVs (group EV), as well as empty defects for control (group E).

**FIGURE 1:**
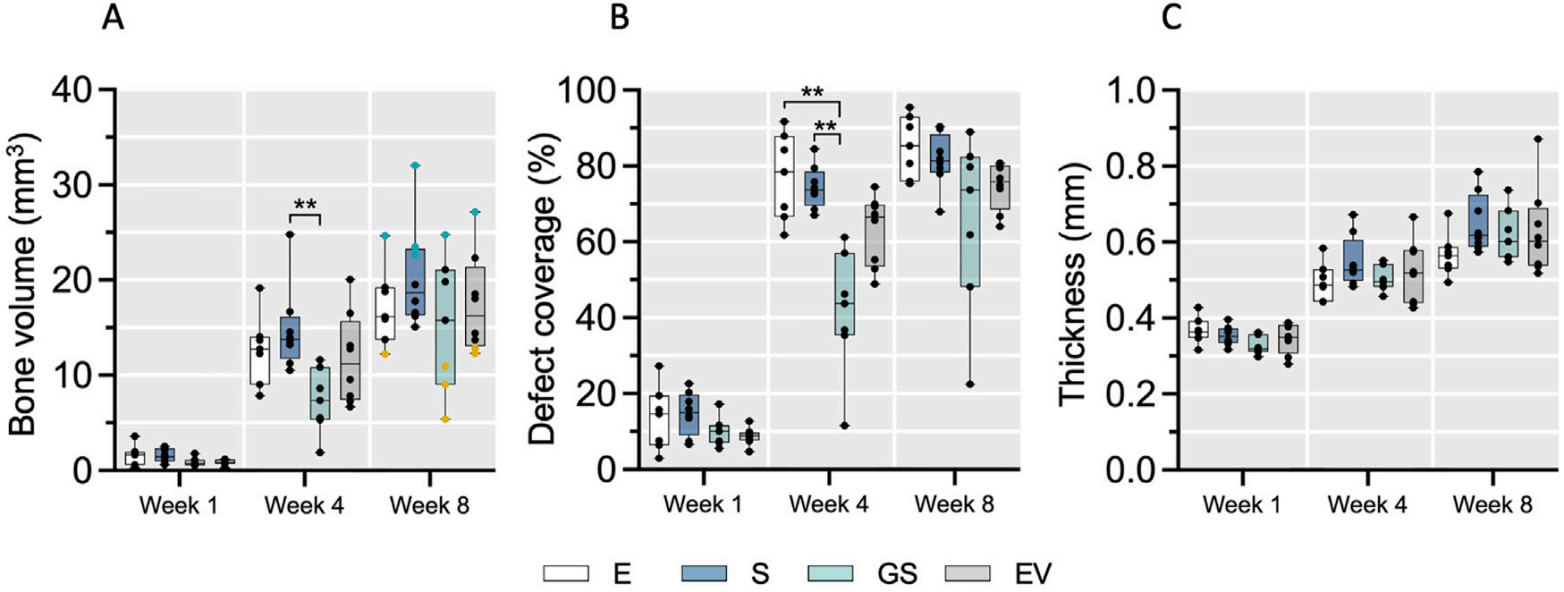
The course of bone defect regeneration. Bone volume **(A)**, bone defect coverage **(B)** and new bone thickness **(C)** were evaluated in the four treatment groups over 8 weeks of healing using microCT. Data are shown as a combination of box plots with median and range and individual values. Statistical differences between treatment groups were determined using a Kruskal–Wallis test with Dunn’s *post hoc* test for multiple comparison, with ** (*p* < 0.01) denoting significant differences. Yellow data points indicate weak regenerators, while turquoise data points indicate strong regenerators. E: empty defect; S: non-augmented CHA scaffold; GS: CHA scaffolds loaded with plasmid encoding for BMP2 and miR-590-5p; EV: CHA scaffold loaded with EVs.

New bone formation initiated in the periphery of the calvaria defect and progressed towards the central part without fully closing the defect area within the observation period (Supplementary Figure S1). Additionally, in some animals, a formation of detached bony islands was observed in the middle of the defect. Quantitative analysis revealed minimal new bone formation (BV) and a defect coverage of less than 20% one-week post-surgery (Figures 1A,B). The strongest increase in both parameters occurred between the first and fourth week after surgery in all treatment groups, followed by a slower increase between weeks 4 and 8. No fully closed defects were observed after 8 weeks. A comparable trend was observed for the calvaria thickness (Figure 1C). Inter-group comparison demonstrated statistical significance only at week 4 for bone volume (S vs. GS; p < 0.01) and coverage (E vs. GS, S vs. GS; p < 0.01), surprisingly indicating that in this model Wharton’s jelly MSC-EVs did not improve bone regeneration over scaffold only, while addition of the non-viral gene transfer strategy seemed to decelerate calvaria bone regeneration at least in the initial 4 weeks. By the final time point (week 8), the median values of bone volume (BV), coverage and calvaria thickness nearly converged for all groups. However, considerable intra-group variability identified animals with strong and weak regeneration within each treatment group. Based on these observed differences, 6 animals exhibiting strong (“good”) regeneration and 6 animals exhibiting weak (“bad”) regeneration were selected (blue and yellow marked animals in Figure 1A, week 8; Supplementary Figure S1, S2) and further assessed histologically and for variations in circulating miRNAs as potential biomarkers for regeneration.

**FIGURE 2:**
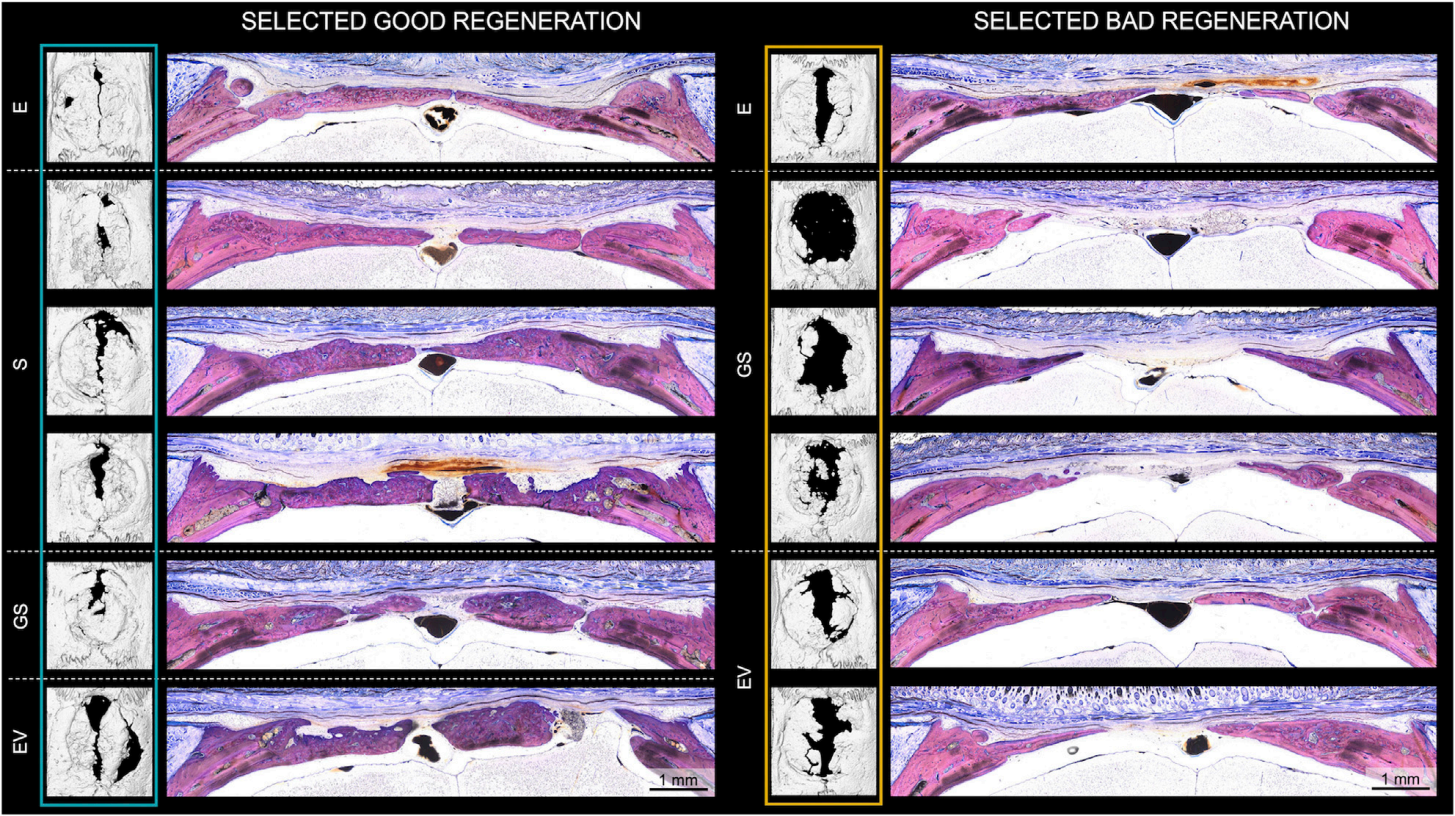
Histological evaluation of bone regeneration in animals exhibiting strong (“good”) and weak (“bad”) regeneration according to microCT analysis. Histological analysis of all 6 selected strong (E, n = 1; S, n = 3; GS, n = 1; EV, n = 1) and weak (E, n = 1; GS, n = 3; EV, n = 2) regenerators after 8 weeks of healing. Undecalcified coronal thin-ground sections were prepared through the center of the defect and stained with Levai-Laczko dye. E: empty defect; S: non-augmented CHA scaffold; GS: CHA scaffolds loaded with plasmid encoding for BMP2 and miR-590-5p; EV: CHA scaffold loaded with EVs.

To evaluate morphological differences between strong (n = 6) and weak (n = 6) bone regeneration, undecalcified coronal thin-ground sections were prepared through the center of the defect. Histologically, new bone formation in strong regenerators (E, n = 1; S, n = 3; GS, n = 1; EV, n = 1) appeared mainly completed with only minor active zones of ongoing osteosynthesis at week 8 (Figure 2; Supplementary Figure S3). These active zones were predominantly found near the defect borders and marked by the presence of osteoblast seams and yet uncalcified osteoid tissue. In all groups including the empty control (E), newly formed bone consisted of a core of woven bone covered with superficial layers of parallel-fibered bone facing inferiorly the dura mater and superiorly the skin. Defects initially treated with non-augmented CHA scaffolds (S) or augmented CHA scaffolds (GS or EV) additionally showed scaffold fibers integrated into the immature woven bone, indicating *de novo* bone formation within the scaffold compartment, which seemed to be subsequently remodeled and replaced by parallel-fibered bone tissue. However, the degree of residual CHA fibers within the bone or as stand-alone fibers colonized by cells in the connective tissue varied considerably among the selected specimens. Two specimens from six chosen strong regenerators showed no evidence of remaining scaffold material 8 weeks post-surgery. The morphological appearance of newly formed bone in CHA treatment groups (S, GS, EV) revealed variations in the ratio between woven and parallel-fibred bone, suggesting different stages within the regenerative process. Unlike the empty control (E), the superficial bone surfaces within these groups displayed irregular patterns rather than a continuous and uninterrupted appearance, indicating resorption as part of the remodeling process.

Weak regenerators (E, n = 1; GS, n = 3; EV, n = 2) (Figure 2 right; Supplementary Figure S3) revealed a similar trend, albeit with decreased bone formation, leaving a broader vascularized area of fibrous soft tissue bridging the remaining defect gap. In comparison to the strong regenerators, weak regenerators treated with CHA groups presented a reduced proportion of integrated fibers in the newly formed bone. Moreover, only minimal amounts of fibers were identified in the soft tissue.

### 3.2 MiRNAs circulating in blood exhibit distinct profiles of up- and downregulation during 8 weeks of bone regeneration

**FIGURE 3:**
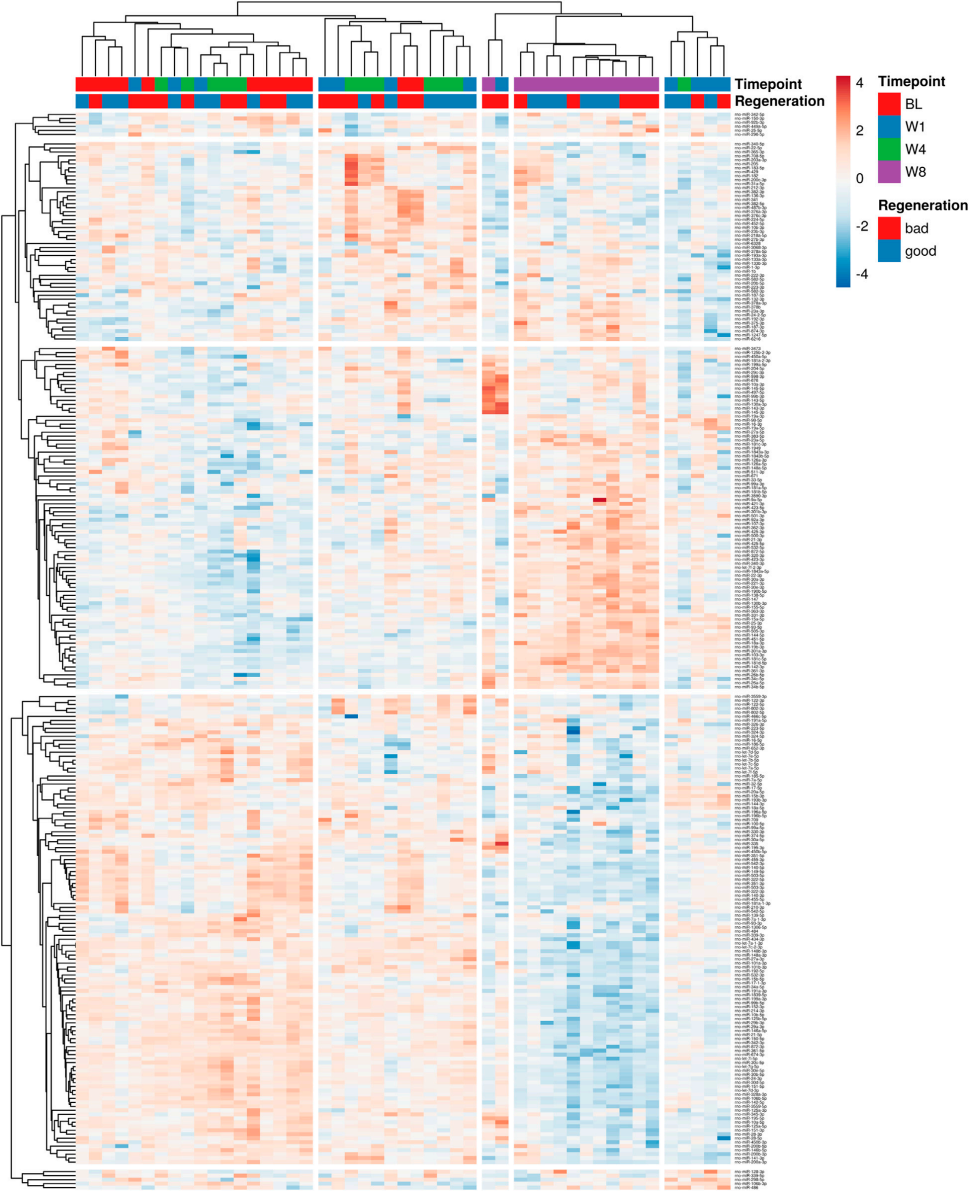
Unsupervised hierarchical clustering analysis of miRNA expression in all samples. Variations across different time points, regeneration stages and individual animals are shown. Different shades of blue denote lower levels of individual miRNA expression, while yellow to red shades indicate higher expression levels. Data used for the generation of heatmap are provided in Supplementary Data File 2.

To identify profiles of miRNAs circulating in the blood associated with bone regeneration, next-generation sequencing was conducted. First, the sequencing runs’ quality was verified based on the consistent detection of spike-in controls (Supplementary Figure S4). The number of miRNAs detected and identified in total as well as the number of miRNAs with read counts above 10 was comparable between the samples (Supplementary Figure S5, data are publicly available at the NCBI GEO archive under the accession ID GSE279239). To visualize the complexity of miRNA expression during bone healing, unsupervised hierarchical clustering analysis of miRNA expression in all samples was conducted (Figure 3). No distinct clustering of samples according to the observed regenerative capacity or time point was observed, albeit samples at week 8 mainly grouped together in one clade.

**FIGURE 4:**
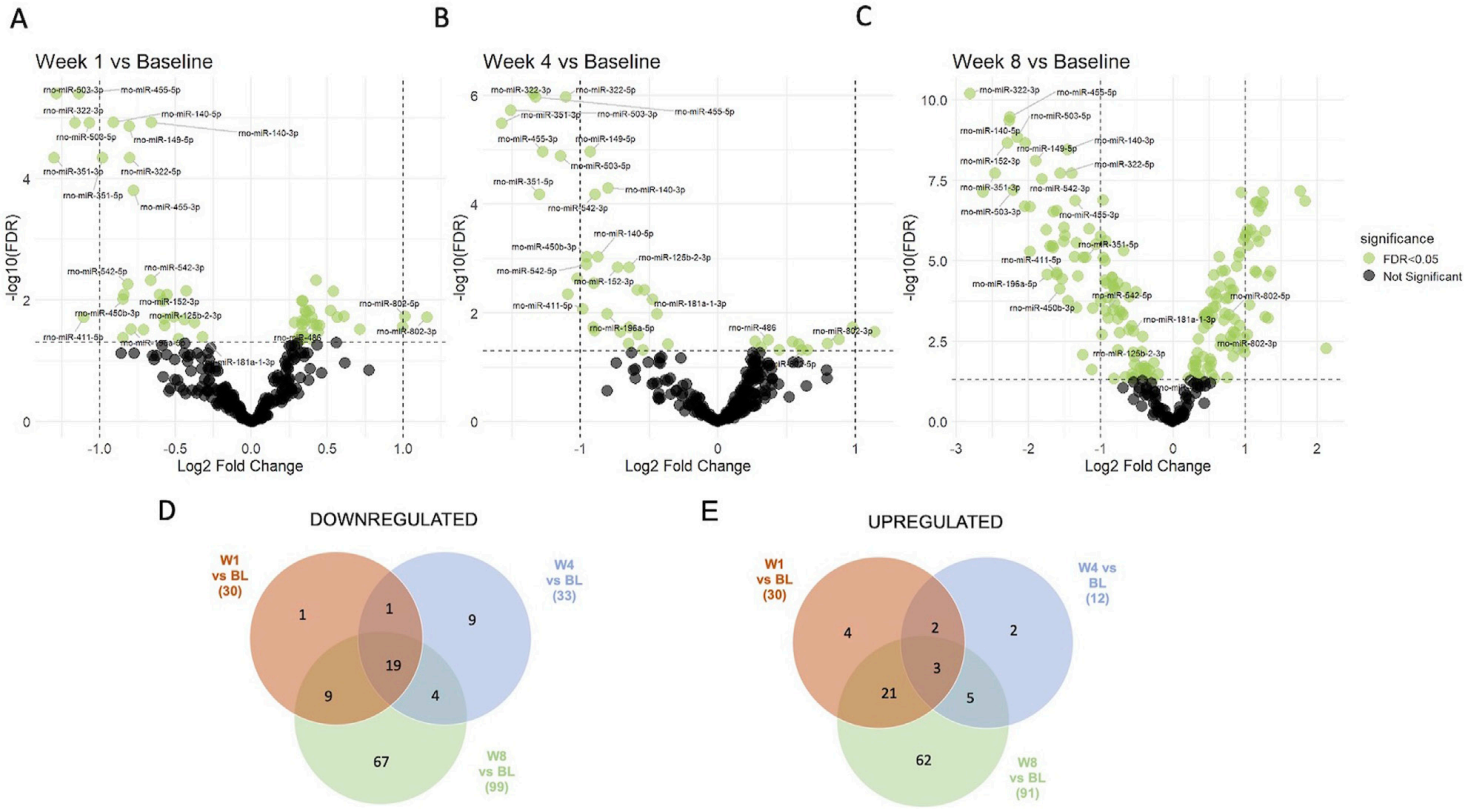
Supervised analysis of miRNA expression at different timepoints compared to baseline. 60 significantly regulated circulating miRNAs (adjusted p-value (FDR <0.05), shown in green color) were identified at week 1 **(A)**, 45 at week 4 **(B)** and 190 at week 8 **(C)** as compared to their baseline expression in all four treatment groups. Venn diagrams show the numbers of miRNAs that were **(D)** downregulated and **(E)** upregulated at different timepoints compared to baseline (BL) and shared between different timepoints. Lists of all regulated/shared miRNAs at the different timepoints are provided in Supplementary Data File 1.

**FIGURE 5:**
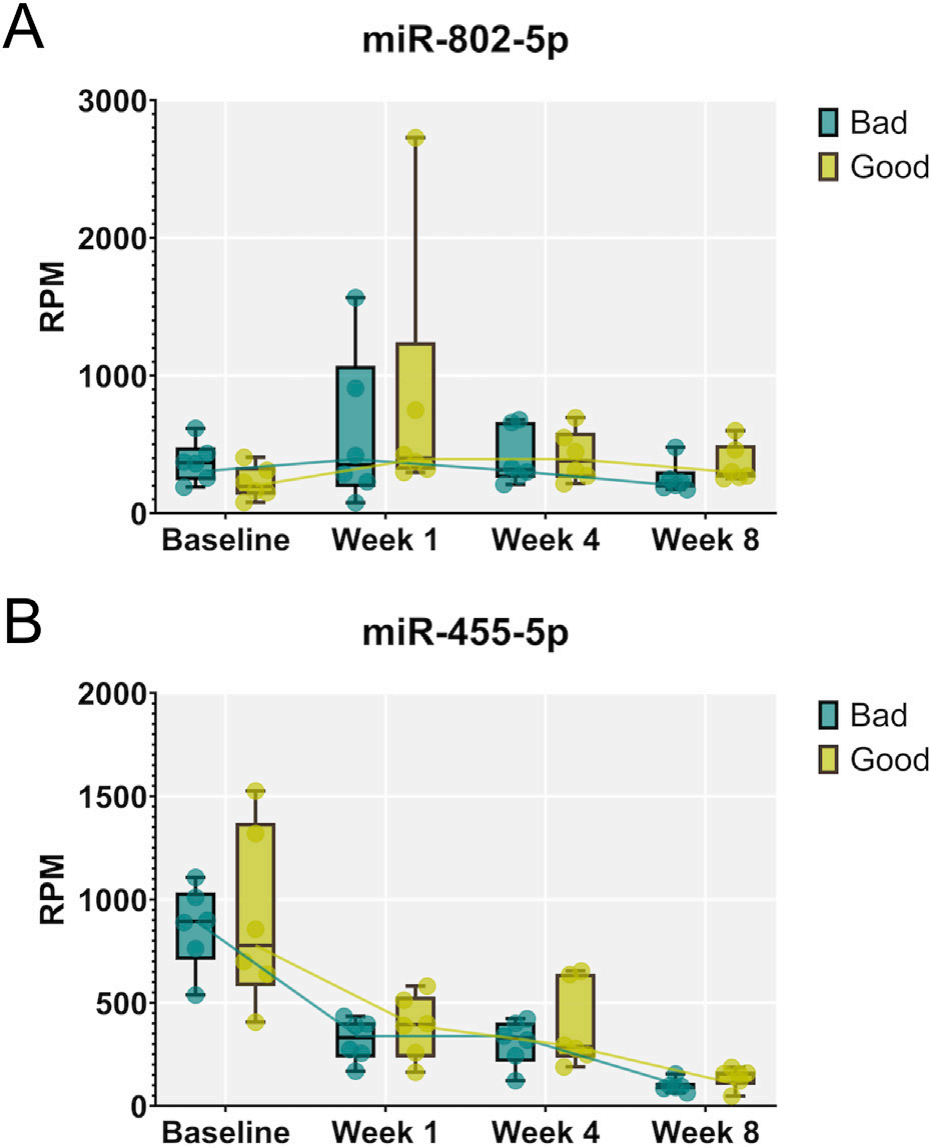
Expression patterns of individual miRNAs that were significantly regulated compared to baseline at all three timepoints of healing. Examples of miR-802-5p, upregulated at all three timepoints **(A)**, and miR-455-5p, downregulated at all three timepoints **(B)**, are shown for animals exhibiting strong (“good”) and weak (“bad”) regeneration. Data are shown as a combination of box plots with median and range and individual values. Yellow data points indicate weak regenerators, while turquoise data points indicate strong regenerators.

Next, supervised statistical tests were applied to identify individual circulating miRNAs that changed in animals irrespective of the treatment group at week 1, 4, and 8 compared to the baseline. Several significantly regulated miRNAs were identified with over 2-fold changed expression level (FDR <0.05) at each of the timepoints (Figures 4A–C). Interestingly, the number of miRNAs significantly different to baseline was the highest after 8 weeks. Furthermore, several miRNAs were identified that were significantly different to baseline at all three timepoints (19 downregulated and 3 upregulated), while others were regulated only at specific time points versus base line (Figures 4D,E, Supplementary Data File 1). Significantly downregulated miRNAs compared to baseline at all three timepoints included miR-125b-2-3p, miR-140-3p, miR-140-5p, miR-149-5p, miR-152-3p, miR-181a-1-3p, miR-196a-5p, miR322-3p, miR-322-5p, miR-351-3p, miR-351-5p, miR-411-5p, miR-450b-3p, miR4553p, miR-455-5p, miR-503-3p, miR-503-5p, miR-542-3p, miR-542-5p, while miR-486, miR-802-3p and miR-802-5p were significantly upregulated at all three timepoints. Individual changes in expression patterns were observed, as represented by two examples, miR-802-5p which was upregulated and miR-455-5p which was downregulated at all timepoints compared to baseline (Figure 5).

### 3.3 Circulating levels of miR-133a-3p and miR-375-3p are significantly different between animals exhibiting strong and weak regeneration

**FIGURE 6:**
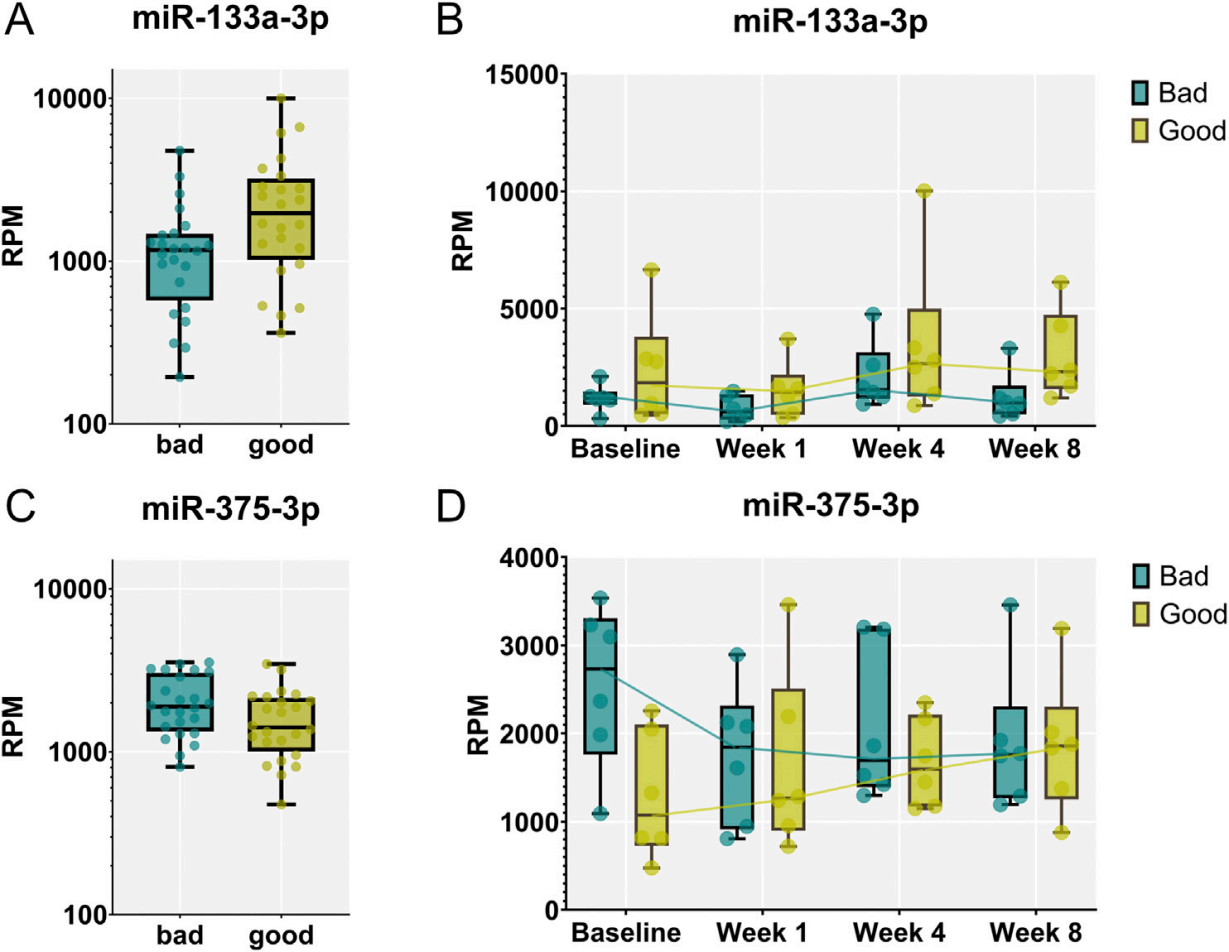
miR-133a-3p and miR-375-3p are differentially expressed between animals exhibiting strong vs. weak regeneration. **(A, C)** Significantly different levels of expression in the blood were found between the two groups of animals analyzing samples from all timepoints. miR-133a-3p **(A)** was significantly up-regulated in strong (“good”) regenerators across all time points (FDR < 0.05) while miR-375-3p **(C)** was identified to be down-regulated. **(B, D)** Expression patterns of miR-133a-3p **(B)** and miR-375-3p **(D)** at different timepoint in animals exhibiting strong and weak regeneration. Data are shown as a combination of box plots with median and range and individual values. Yellow data points indicate weak regenerators, while turquoise data points indicate strong regenerators.

To identify miRNAs circulating in the blood that differ between rats exhibiting strong versus weak calvaria defect regeneration, we compared the samples of the corresponding animals across all time points (FDR <0.05). Indeed, we identified miR-133a-3p to be significantly upregulated (Figures 6A,B), while miR-375-3p was found to be significantly downregulated in strong regenerating animals over all timepoints studied (Figures 6C,D). Of note, these 2 miRNAs already differed significantly at baseline–shortly before the calvaria defect was generated–suggesting that they might have prognostic value to identify strong versus weak regenerative capacity before wounding.

### 3.4 miRNA target prediction for mir-133a-3p and mir-375-5p

**FIGURE 7:**
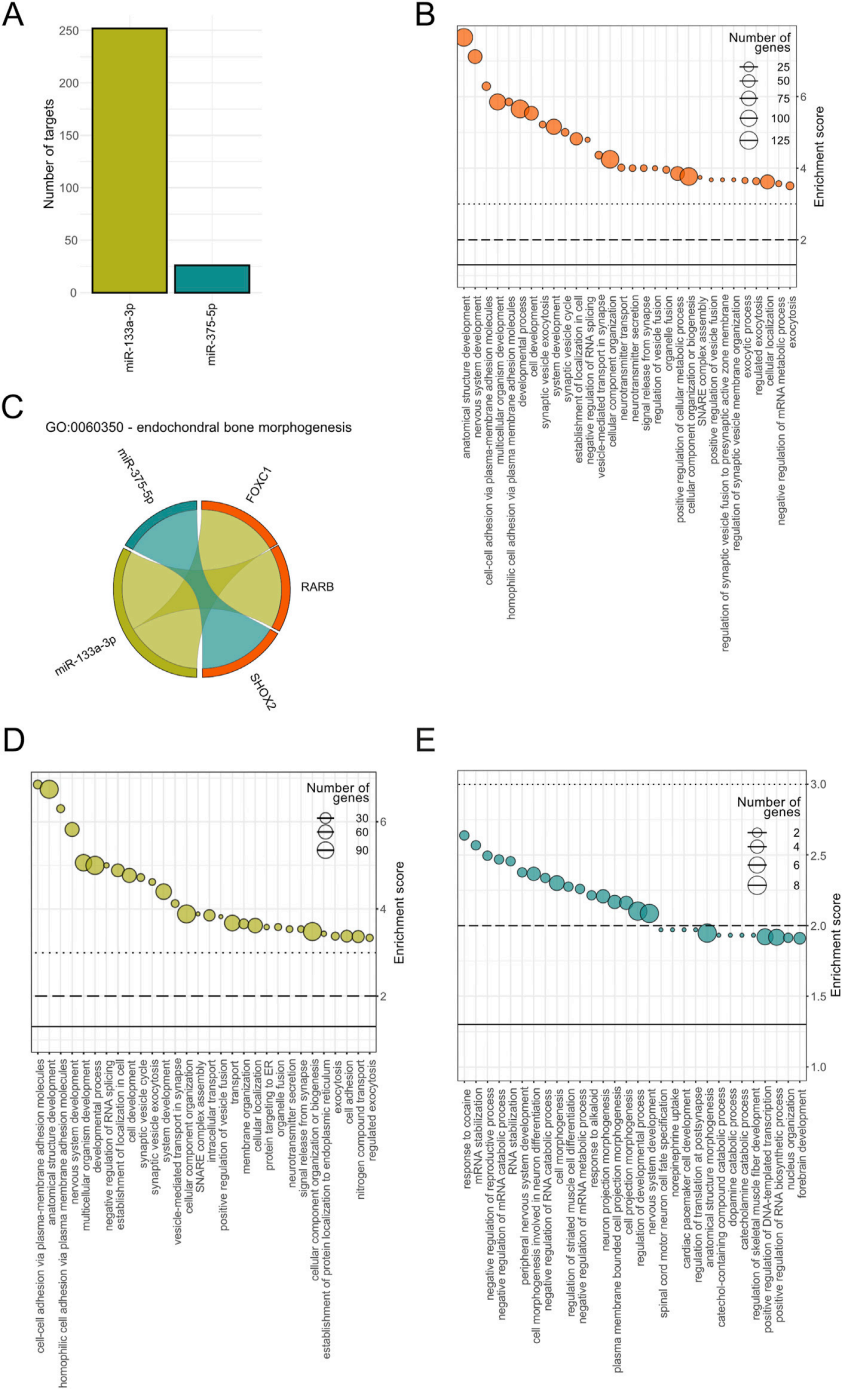
Functional enrichment analysis of predicted mRNA targets for hsa-miR-133a-3p and hsa-miR-375-5p. **(A)** Number of target genes for miR-133a-3p (green) and miR-375-5p (turquoise). **(B)** Top 30 significantly enriched Gene Ontology (GO) terms associated to biological processes (BP) based on combined miRNA targets. Enrichment scores (-log10(P value) as well as the number of target genes per pathways are displayed for each BP, on the y-axis and size range of the dots, respectively. **(C)** GO term specific interaction diagram for GO:0060350 - endochondral bone morphogenesis. Interactions between miR-375-5p and SHOX2 and miR-133a-3p with FOXC1 and RARB are color coded in turquoise and green, respectively. Top 30 significantly enriched BPs for has-miR-133a-3p **(D)** and for hsa-miR-375-5p **(E)** with the number of predicted target genes shown following the same format as in **(B)**.

The target prediction analysis identified 252 mRNA targets for hsa-miR-133a-3p, 0 targets for hsa-miR-375-3p and 26 mRNA targets for hsa-miR-375-5p (Figure 7A). The predicted targets were filtered to ensure inclusion only if they were identified by at least three databases, resulting in a refined and reliable dataset.

Gene Ontology (GO) analysis revealed 375 significantly enriched biological processes based on combined miRNA targets. The top 30 BPs ranked on P value included processes linked to development (anatomical structure, nervous system, multicellular organism, developmental process), cell adhesion and synaptic vesicles (Figure 7B). Notably, endochondral bone morphogenesis was found to be significantly enriched including two targets associated to miR-133a-3p, retinoic acid receptor beta (RARB) transcription factor and forkhead box C1 (FOXC1) transcription factor, and one target interaction for miR-375-5p, i.e., short-stature homeobox 2 (SHOX2) (Figure 7C). Isolated enrichment analysis for miR-133a-3p specific targets showed top terms overlapping with the combined GO analysis including mainly biological processes associated to synaptic vesicles (Figure 7D). The number of annotated genes involved in these processes ranged from 30 to 90. For miR-375-5p, the enriched pathways included catabolic associations and nervous system-related biological processes, with the nervous system-related processes also appearing in the combined analysis (Figure 7E).

## 4 Discussion

Bone regeneration is a widely studied topic and encompasses a large variety of treatment approaches involving combinations of biomaterials, osteo-inductive factors and/or therapeutic cells. In the present study, we attempted to enhance calvaria defect regeneration by augmenting CHA scaffolds with therapeutic pDNA and MSC-EVs. However, while the pDNA group delayed regeneration slightly but significantly, no significant effects were found comparing MSC-EVs with non-augmented scaffolds or empty defects. This suggests no adverse effects of MSC-EVs locally applied for augmenting CHA scaffolds, but also shows a large variability in the regenerative capacity of individual animals. We therefore decided to evaluate levels of miRNAs circulating in the blood over the course of defect healing, to test whether these would differ between animals exhibiting strong versus weak regeneration. Six animals exhibiting best and six animals exhibiting worst regeneration according to µCT data were selected for miRNA analysis. We identified a number of miRNAs exhibiting individual expression patterns during the course of defect healing, potentially providing surrogate, minimally-invasive markers of bone healing. Furthermore, we found that miR-133a-3p was upregulated, while miR-375-3p was downregulated in animals exhibiting strong regeneration as compared to animals exhibiting weak regeneration across all timepoints evaluated, importantly even before injury. Such prognostic biomarkers might help to identify individuals at risk of low bone regenerative capacity or even of developing non-unions.

It is well recognized that miRNAs impact bone development and homeostasis through regulating the growth, differentiation and functional activity of cells that constitute bone tissue (Lian et al., 2012; Chen et al., 2017; Feng et al., 2018; Zhao et al., 2018). For instance, miRNAs which inhibit proliferation and osteoblast differentiation, including miR-125, miR-133 and miR-138, were found to be downregulated in bone morphogenetic protein-induced osteogenesis (Lian et al., 2012). Others, such as miR-140, can positively impact bone development by promoting chondrogenesis. However, it remains to be elucidated which miRNAs participating in bone homeostasis and repair are shed into the blood and could be used as “liquid biopsy” to monitor the progress and the different stages of bone regeneration.

In the current study, miR-486, miR-802-3p and miR-802-5p were found to be upregulated at all three timepoints (weeks 1, 4 and 8) compared to their baseline levels in the blood prior to surgeries (week 0). Although there is a scarcity of literature about miR-802 being involved in bone formation, there is increasing evidence showing that miR-802 promotes the progression of osteosarcoma through targeting p27 and activating PI3K/AKT pathway (Gao et al., 2022). Similarly, there are no prior studies that we would be aware of linking elevated blood levels of miR-486 to bone repair. However, it has previously been reported that exosomes from bone marrow stromal cells carrying miR-486-5p have a protective effect on myocardium ischemic injury by suppressing PTEN expression, activating the PI3K/AKT signaling pathway, and subsequently inhibiting the apoptosis of injured cardiomyocytes (Sun et al., 2019). Interestingly, suppression of PTEN by a variety of different miRNAs and concomitant activation of PI3K/AKT signaling has been found to advance bone regeneration in various models by supporting osteogenesis (Zheng et al., 2019; Zhao et al., 2022) and vascularization (Yang et al., 2022), while suppressing osteoclastogenesis (Zhao et al., 2015).

On the other hand, among the 19 miRNAs significantly downregulated at all three timepoints compared to pre-surgery baseline were miR-455-3p and miR-455-5p. MiR-455-5p has been reported to attenuate neuronal apoptosis in spinal cord ischemia-reperfusion injury model (Liu et al., 2022) and to show anti-inflammatory effects (Torabi et al., 2019). However the role of miR-455-5p in bone regeneration remains to be further elucidated (Liu et al., 2022).

It was prior shown that decreased miR-144-3p affects osteoclast formation, proliferation and apoptosis and therefore makes it an important marker for mediating bone homeostasis (Wang et al., 2018). In addition, downregulation of miR-144-3p induces osteogenesis (Yang et al., 2023). In contrast, it is low in plasma of osteoporosis patients and acts on osteoclastogenesis (Wang et al., 2018). However, miR-114-3p was not among significantly downregulated miRNAs in the current study.

We next investigated whether miRNA expression levels could differentiate between animals exhibiting strong and weak bone regeneration. In this analysis, samples collected prior surgery and at all three timepoints for all six selected animals were included for each group. Interestingly, we identified miR-133a-3p to be significantly upregulated in strong regenerators. Interestingly, in the plasma of patients carrying plastin 3 (PLS3) mutation who are suffering from severe childhood-onset osteoporosis, this miRNA was found to be downregulated (Mäkitie et al., 2020). In a rat model for rotator cuff rupture regeneration, where we treated one group with a single dose of zoledronic acid, miR-133a-3p was upregulated upon treatment, which resulted in better regeneration. Zoledronic acid treatment influenced circulating miRNAs, including miR-133a-3p, which was linked to better muscle regeneration. (Rivas et al., 2021; Schanda et al., 2022). These findings would indicate serum or plasma levels of miR-133a-3p as predictive for better regeneration, even though miR-133a-3p is mainly connotated as a muscle specific miRNA (Margolis et al., 2017). However, in the bone context, miR-133a-3p has previously been reported to negatively regulate osteogenic differentiation by targeting MAPK in a mouse model of age-dependent osteoporosis (Zhong et al., 2024), while bone morphogenetic protein-induced osteogenesis resulted in decreased levels of miR-133 in osteoblastic cells (Li et al., 2008; Lian et al., 2012).

One other miRNA, miR-375-3p, was significantly downregulated in strong regenerating animals. miR-375-3p has been reported to promote osteogenic differentiation of human adipose-derived mesenchymal stromal cells and bone regeneration (Chen et al., 2017; 2019). In contrast, Sun et al. (Sun et al., 2017) reported that miR-375-3p negatively regulated osteogenesis by targeting and decreasing the expression levels LRP5 and β-catenin, and Du et al. (2015) similarly found that miR-375 overexpression inhibited key osteoblast markers. It remains to be elucidated, how the relatively increased levels of miR-133a-3p and decreased levels of miR-375 in animals exhibiting strong versus weak regeneration as identified in our study are mechanistically linked with the reported roles of these miRNAs in osteogenic differentiation and the progress of bone healing process.

Our target prediction analysis identified 252 mRNA targets for miR-133a-3p and 26 mRNA targets for miR-375-5p. GO analysis indicated that miR-133a-3p targets were associated with biological processes such as cell adhesion and development, while miR-375-5p was linked to catabolic and nervous system-related biological processes. Interestingly, combined GO analysis revealed endochondral bone morphogenesis as one of the significantly enriched biological processes relevant to bone regeneration, with transcription factors RARB and FOXC1, and SHOX2, as targets. RARB has previously been identified in gene regulatory network of osteosarcoma (Luo et al., 2013), whereas FOXC1 (together with FOXC2) was shown to direct hypertrophic chondrocyte maturation and function toward primary ossification center formation and osteoblast recruitment (Almubarak et al., 2024). SHOX2 is required to form the proximal bones of the limbs, the humerus and femur in the mouse, whereas deficiencies of human gene cause the short stature in different syndromes (Vickerman et al., 2011). These findings further support the potential roles of these miRNAs in bone healing and suggest their involvement in biological processes critical to regeneration. Identified differences in the number of involved target genes as well as the total number of significantly enriched biological processes between the two microRNAs underscore the differences in their functional impact. These findings indicate that miR-133a-3p likely exhibits a broader range of regulatory targets compared to miR-375-5p, reflecting potential differences in their biological roles and pathway involvement.

The present study has several limitations. Firstly, the study was originally designed to compare options to augment bone repair using CHA scaffolds in the calvaria drill hole model. However, due to the significant variability within the treatment groups and the empty defect control, we chose to focus on 6 animals with the strongest and 6 animals with the weakest regeneration outcomes to identify circulating miRNAs that may correspond to differences in regenerative capacity, both cross-sectionally and longitudinally. Therefore, the number of animals selected out of the four groups was not fully symmetric. Further studies are needed to evaluate whether the miRNAs identified here can be used as predictive factors of successful bone regeneration, and to elucidate their underlying mechanisms of action. Furthermore, clinical relevance of our findings remains a matter of speculation that would require appropriate clinical study validation. Nonetheless, our results are among the first to point towards using circulating miRNAs as indicators of the regenerative potential of single animals after bone defects.

## Data Availability

Small RNA-sequencing raw data have been deposited at the NCBI Gene Expression Omnibus (GEO) archive under the accession ID GSE279239 (https://www.ncbi.nlm.nih.gov/geo/). All other datasets are included in the manuscript figures and supporting files.

## Supplementary material

SUPPLEMENTARY FIGURE 1 microCT overview of bone regeneration in rat calvaria defects. Radiological images show the course of healing 1, 4 and 8 weeks after the surgeries for empty defects and defects treated with non-augmented CHA scaffolds or augmented CHA scaffolds (gene-activated or extracellular vesicle-loaded). Bone formation initiated at the defect margins and progressed towards the central part without fully closing the defect area within 8 weeks. Turquoise and yellow dashed rectangles indicate strong and weak regenerators chosen for further analysis.

SUPPLEMENTARY FIGURE 2 Bone regeneration parameters for selected individual animals showing strong and weak regeneration. Bone volume, bone defect coverage and new bone thickness are shown for individual animals over 8 weeks of healing. Yellow data points indicate weak regenerators, while turquoise data points indicate strong regenerators.

SUPPLEMENTARY FIGURE 3 Histological evaluation of bone regeneration in animals exhibiting strong vs. weak regeneration. Representative histological subregions at higher magnification show examples of bone regeneration in empty defects and defects treated with non-augmented or augmented CHA scaffolds in animals exhibiting strong (top) and weak (bottom) regeneration. Histological analysis of animals exhibiting strong regeneration after 8 weeks revealed primarily completed new bone formation with only limited active zones, mainly concentrated near the defect margins and characterized by osteoblast seams and osteoid tissue, with some exceptions. Newly formed bone comprised a blend of woven and parallel-fibered bone. Animals treated with one of the different CHA groups (S, GS, EV) showed new bone formation within the scaffold compartment. Amounts of stand-alone fibers were also observed in the soft tissue. A comparable morphological trend was observed between strong regenerators and animals exhibiting weak regeneration, albeit with reduced bone formation and a broader vascularized layer of fibrous soft tissue bridging the remaining defect area. WB: woven bone; PFB: parallel-fibered bone, S: soft tissue; black arrow: osteoblasts; yellow arrow: osteoid; black and white asterisks: CHA fibers (black: integrated in bone; white: integrated in soft tissue); E: empty defect; S: non-augmented CHA scaffold; GS: CHA scaffolds loaded with plasmid encoding for BMP2 and miR-590-5p.

SUPPLEMENTARY FIGURE 4 Recovery of spike in RNA from the RNA sequencing samples. A comparable efficiency of RNA isolation and detection was found between the analyzed samples using three spike-in controls (UniSP.2,.4,.5).

SUPPLEMENTARY FIGURE 5 Number of total miRNAs and miRNAs detected over 10 times in sequencing samples. A comparable number of miRNAs was found between the analyzed samples.

SUPPLEMENTARY DATA FILE 1 Lists of differentially regulated miRNAs (up- and downregulated). miRNAs regulated at the three different timepoints of healing vs. baseline, presented in Venn diagrams in Figures 4D,E are listed.

SUPPLEMENTARY DATA FILE 2 miRNA expression data used for the generation of unsupervised hierarchical clustering heatmap in Figure 3.
