# Supplementary figures and images for: Circulating miRNAs are associated with successful bone regeneration
Source: Front Bioeng Biotechnol. 2025 Mar 28;13:1527493. doi: 10.3389/fbioe.2025.1527493 (PMC11985807; doi:10.3389/fbioe.2025.1527493)

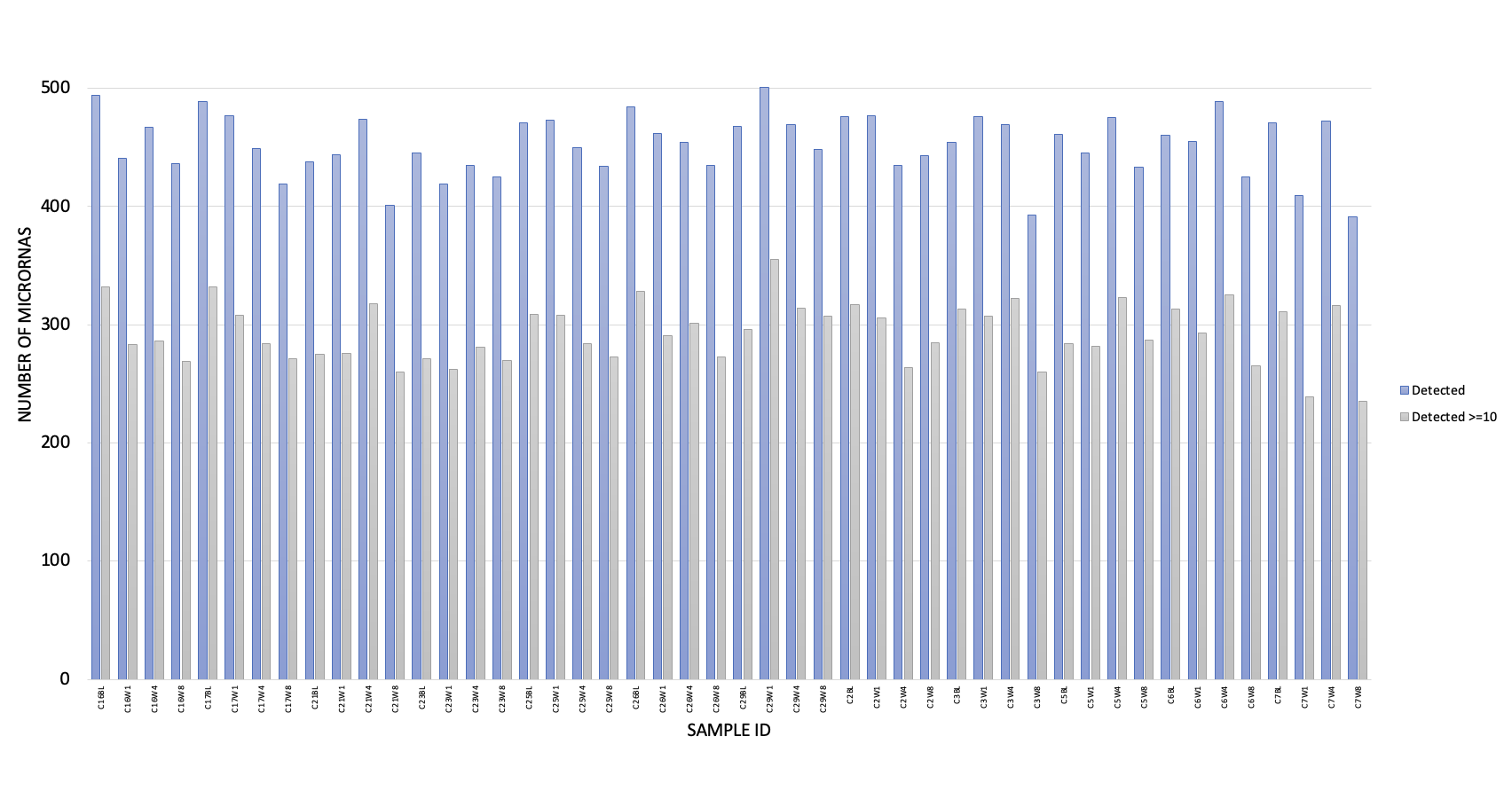

Supplement: Supplementary file 2 [file Image5.TIFF]

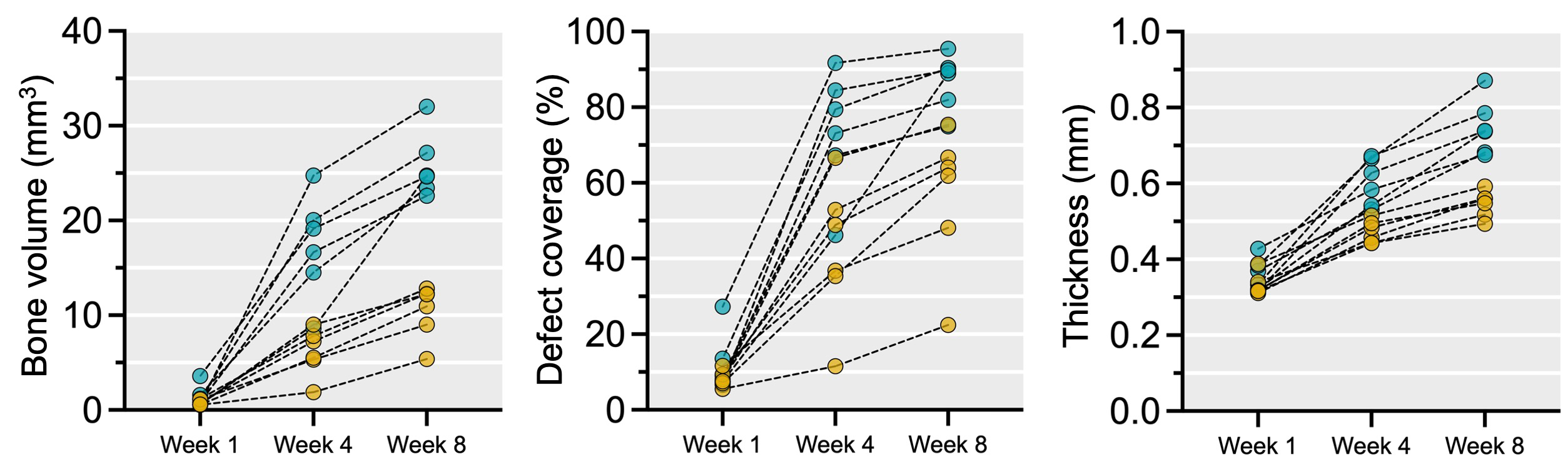

Supplement: Supplementary file 3 [file Image2.TIF]

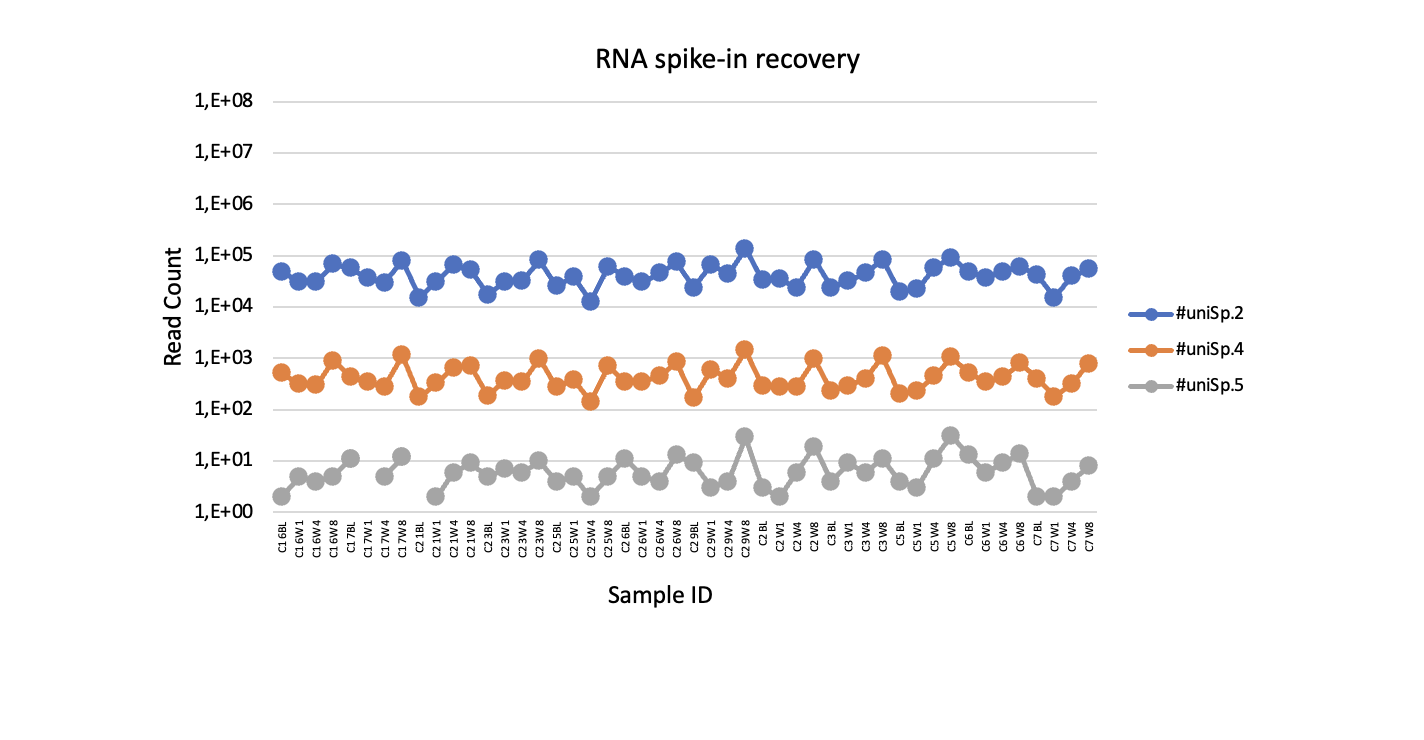

Supplement: Supplementary file 5 [file Image4.TIFF]
